# Evaluation of the Quality of Life in Epileptic Children of Shiraz, Southern Iran

**Published:** 2020

**Authors:** Pegah KATIBEH, Soroor INALOO, Peyman JAFARI, Fahimeh FATTAH, Samaneh MAZLOOMI

**Affiliations:** 1 Department of Pediatric Neurology, Shiraz University of Medical Sciences, Shiraz, Iran; 2 Department of Biostatics, Shiraz University of Medical Sciences, Shiraz, Iran; 3 Department of Pediatrics, Shiraz University of Medical Sciences, Shiraz, Iran

**Keywords:** Epilepsy, Quality of life, KIDSCREEN-27

## Abstract

**Objectives:**

People suffering from chronic diseases like epilepsy are highly prone to debilitating changes in factors that affect the quality of life (QOL) such as physical capacity, self-esteem, relationships with others and fulfillment of their daily life activities. This study attempted to evaluate QOL in children with epilepsy in Shiraz, Southern Iran.

**Materials & Methods:**

Epileptic patients admitted at the epilepsy clinic of Shiraz University of Medical Sciences with no first time episode of seizures in the previous six months and no febrile-seizure were included in the study. The patients were evaluated using the standard KIDSCREEN-27 questionnaire. Data were analyzed using the statistical software SPSS 21 along with Man Whitney and Chi-square tests, and were reported in terms of descriptive statistics. The significance level was considered less than 0.05.

**Results:**

In this case-control study, 229 children with epilepsy were compared with a control group of 400 normal individuals. The mean age was 12.44±3.16 and 12.10±2.69 years in the case and control groups, respectively. The tonic-clonic seizure had the highest prevalence. Moreover, male gender, older age and more seizures per year were associated with lower QOL. Overall, epileptic children had significantly lower QOL compared to the controls.

**Conclusion:**

Epileptic children have an overall lower QOL while factors such as old age, male gender, and high number of seizures per year reduce QOL in epileptic patients.

## Introduction

Epilepsy is a brain disorder characterized by chronic susceptibility to experiencing epileptic seizures in affected individuals. An epileptic seizure is a transient occurrence of signs and/or symptoms due to abnormal synchronous neuronal activity in the brain (1). At any given time, it is estimated that 50 million individuals worldwide are diagnosed with epilepsy (2). In Iran, the prevalence of epilepsy is reported to be as high as 1.8%; however, epidemiologic data are limited on the young population in Iran (3). 

Regardless of physical complications of the disease and those associated with drug side effects, many studies on the quality of life (QOL) in epileptic patients have highlighted the risk of developing a number of socio-cultural and psychological problems in affected individuals, such as lower self-esteem and higher levels of anxiety and depression (4,5). Moreover, epileptic patients are more prone to problems in relation to educational achievement (6,7,8) and social withdrawal and isolation (9,10). When an individual has a chronic condition, for which a total cure is not expected, QOL is considered an important outcome measure for healthcare (11).

In the case of pediatric epilepsy, seizures are more complicated and need more elaborate concerns, due to the very demanding nature of childhood period and the high prevalence of the disease among children, with a prevalence rate of approximately 3-4 per 1,000 children (12,13,14). Epilepsy can have undeniable debilitating effects on children’s well-being and QOL (15,16,17). This, in turn, can have severe effects on the development of a healthy character during childhood (18,19,20), which is directly influenced by development of a successful relationship with peers and having appropriate levels of independency. Thus, handling this disease can be highly challenging for both parents and their affected children.

QOL has been defined as the "subjective evaluation of individuals about the quality of their lives as it relates to their own personal expectations" (21). Therefore, in any explanation on what variables are important to children's QOL, the epileptic child would be the most suitable referee for making such an appraisal. However, based on several reviews on QOL measures in pediatric epilepsy, the use of the affected child's own report has not been dominant in these studies (11,22). 

To the best of our knowledge, there have been few studies about epileptic patients’ QOL undertaken in the Middle Eastern countries (23), with only one study specifically targeting the Iranian population (24). The overall aim of the present study, therefore, was to assess the impact of epilepsy and its treatment on the daily lives of epileptic children in Iran and also to compare and contrast experiences of these children with those of their healthy counterparts in Shiraz, Iran, based on the self-report KIDSCREEN27 questionnaire. The Kidscreen-27 is a well-validated, short, multidimensional measure to assess the health-related QOL in children and adolescents. It has five scales including (1) physical well-being, (2) psychological well-being, (3) autonomy and parents, (4) social support and peers, and (5) school environment (25). The KIDSCEERN27 questionnaire has already been translated into Farsi by the Kid-screen Group and further subdivided to include 27 subscales, covering various perspectives of the child's mental and physical health status. Moreover, the reliability of the questionnaire has been confirmed in several studies on Iranian children (25,26). This work is novel because it relies on both children and their parents' self-report and also on standard measures to assess the health-related QOL in Iranian children with epilepsy. To the best of our knowledge, there has been no such study conducted on epileptic children’s QOL in Iran. 

## Materials & Methods

**Inclusion and exclusion criteria**

The patients in this study were recruited from epileptic children admitted at the Emam-Reza Hospital as the epilepsy center in Shiraz, Southern Iran, from April 2013 to April 2014. . Ethics approval was granted by the hospital research ethics committee for each of the participating programs. Further, informed consent was obtained from all caregivers and the child's assent was obtained when appropriate. Inclusion criteria were as follows: age range of 8 to 18 years at the time of enrollment, ability to report in Farsi and no experience of first time seizures in the previous six months. Children with febrile-seizure were excluded from the study. In such cases, seizure is usually triggered during the age of 6-60 months due to reasons other than CNS infections or electrolyte imbalances and the child has no previous history of seizures without fever. Children with mental retardation and speaking disorders were also excluded from the study.

The control group members were randomly selected from different schools in the quadruplet districts of Shiraz. 

**Parent and child self-report questionnaires**

The Emam-Reza Hospital is the main epilepsy center in the south of Iran. Thus, the target population of the study can be regarded as the representative of Southern Iranian population. 

In this study, the KIDSCEERN27 questionnaire was used, which psychometrically assesses 8-18-year-old epileptic children. The KIDSCEERN27 questionnaire was already translated into Farsi by the Kid-screen Group (26). The instrument consists of 27 items divided into seven subscales which are respectively about the child's physical well-being, their psychological well-being, their relationship with parents, autonomy, social support, their interaction with peers and finally school environment. The children and their parents filled out the self- and proxy-report of the KIDSCREEN27 questionnaire, independently. All the children's self-report measures were scored positively such that a higher score represented more of the desirable trait. The validity and reliability of the questionnaire have been approved in other studies on Iranian population (26,27).

**Statistical analysis**

The obtained empirical data were analyzed using the statistical software SPSS 21 along with descriptive statistics (arithmetical mean and standard deviation), the Mann-Whitney test (due to inequality of data variance), and the Chi-square test. The significant level was set at p<0.05. 

## Results

In this research, 229 epileptic patients were compared with 400 normal cases. The mean age of children in the case and control groups was 12.44 ± 3.16 and 12.10 ± 2.69 years old, respectively. No significant difference was observed between the ages of the patients and controls (p>0.05). Moreover, there were no significant differences in terms of gender between the two groups (p>0.05), with 102 and 219 boys being respectively included in the groups of patients and controls. The most common type of seizure among the patients was tonic-colonic seizures, affecting 103 (44.97%) of the patients (Table 1). About 47% of the patients were treated with two or more drugs whereas 53.2% of them received mono-therapy. In 70.74% of the patients, the disease was controlled and no seizures were reported. Of the patients, 22.7% had one or two seizures in a year and 6.55% experienced more than two seizures per year.

Analysis of the data from the KIDSCREEN27 questionnaires showed the following results:

In terms of physical well-being, there was no significant difference between the two groups based on the reports of the parents (P = 0.218) and their children (p = 0.412). However, concerning all the other six criteria (psychological well-being, relationship with parents, autonomy, social support, peers and school environment), members of the control group (both parents and their children) responded more positively to the questionnaire. Reports of children from the control group showed that they were significantly more satisfied with their parents and more enjoyed their spare time, as compared to the patients. Moreover, the children were coping better with their peers, had less trouble concentrating at school, and were more productive in learning processes at school. The data obtained from questionnaires submitted by the parents also revealed similar results (Table 2).

In addition, the results showed that age was a discriminative factor for the QOL of epileptic patients, with QOL decreasing as the patient's age increased. However, in terms of physical well-being, the children's age had no significant effect on their report. The results also revealed that the girls significantly performed better in all of the questionnaire's subscales except those related to relationship with peers and school environment, as compared to the boys.

Controlling the disease was another determining factor for the child's QOL. As the number of seizures per year decreased, the patients’ QOL significantly increased. However, even among patients whose disease was fully controlled, QOL was significantly lower compared to the controls. Although patients who received a single anti-seizure drug had better QOL scores compared to those receiving two or more drugs, the difference was not significant (p > 0.05).

It was also found that longer treatment of a patient with anti-seizure drugs led to lower QOL scores. However, drugs' effect was insignificant on the children's physical well-being and the quality of their spare time (p > 0.05). 

## Discussion

Epilepsy is one of the most common and serious neurological diseases, affecting both adults and children. The disease is associated with non-deniable life limiting complications that may severely affect patients' well-being in daily life and thus significantly change their QOL (28). However, despite many studies conducted on QOL in adults and children with epilepsy, very few studies have investigated QOL through the direct exploration of patients' own views and reports (11,29).

According to the results, except for physical health, the epileptic children showed significantly lower scores on psychological well-being, relationship with parents, autonomy, social support, interaction with peers and finally school environment. The reports of their parents also followed the same pattern.

The data also demonstrated that by increasing the age, QOL scores significantly decreased in all the measured criteria. Arya and his colleagues conducted a similar study on the QOL of children suffering from epilepsy. In agreement with our results, they found that older children were more prone to anxiety and lower self-esteem (30). This may be due to the higher self-awareness of these children regarding their disease or their further involvement in social issues as the age increases (31,32). Drugs' side effects can also be an effective factor considering the fact that older children have longer experience of anticonvulsants' side effects (33). 

It was also realized that the girls significantly scored higher in different parts of the questionnaire compared to the boys. This may arise due to the higher competitive nature of boys, which increases their expectation to indulge and succeed in social activities. Although several previous studies confirm our result (33,34), the results of two recent studies disprove it, suggesting that the gender of patients has no significant effect on their QOL. 

The findings of the current study indicated that the increasing number of seizures per year had a worsening effect on the patients’ QOL. This is in contrast with the result of many studies considering the number of seizure episodes per year (30,33,36,37). Moreover, no significant association was observed between seizure type and the patients’ QOL. Although the results of the study by Arya and colleagues are in agreement with our results, many other studies suggest that seizure type is a contributing factor (33,36,38).

In this study, reports of both parents and their children were considered on the epileptic child’s QOL. Accordingly, it was observed that reports of both parents and children on QOL items had similar patterns. However, in terms of physical well-being, psychological well-being and school environment, scores of the parents were lower than those of their epileptic children (Table 3). In two separate studies, Ronen and Teylor found that children and parents' reports had similar patterns. They concluded that parents may be reliable proxies for their children (39, 40). However, in a review by McEwan and colleagues, it was suggested that parents and children may have different perceptions of QOL, which is especially important for younger children. Children appear to be more concerned regarding several items like restrictions of activities, loss of independence and difficulties with peer relationships, although, in general, positive relationships with families have been reported (11). Nevertheless, studies using proxy informants have highlighted issues such as educational attainment and cognitive difficulties (11). Reports of parents are especially important in cases that the child cannot respond independently. However, it appears that viewpoints of both parents and children should be considered concurrently.

**Table 1:** The distribution of different types of seizures among the patients

**Female**		**Male**		**Total**		**Seizure** **Type**
**Number**	**(%)**	**Number**	**(%)**	**Number**	**(%)**	
36	15.72	42	18.34	78	34.06	**Partial**
51	22.27	52	22.70	103	44.97	**GTC**
3	1.31	14	6.11	17	7.42	**Absence**
5	2.18	9	3.93	14	6.11	**Myoclonus**
7	3.05	10	4.36	17	7.41	**Other**
102	44.53	127	55.46	229	100	**Total**

*GTC: generalized tonic clonic

**Table 2:** Scores of the patient and control groups from the KIDSCREEN27 questionnaire

P-value*	**Controls score**	**Patients score**	** Section**	
	**Mean ± SD**	**Mean ± SD**		
0.412	51.67 ± 11.60	50.94 ± 10.79	**Physical well-being**	**Child's View**
0.000	45.37 ± 10.60	41.92 ± 10.43	**Psychological well-being**	
0.000	48.73 ± 9.87	44.21 ± 4.01	**Autonomy and relationship with parents **	
0.000	44.86 ± 9.35	41.18 ± 7.75	**Social and peer relationship**	
0.000	52.80 ± 9.80	46.40 ± 5.59	**School environment**	
0.018	50.80 ± 10.94	49.98 ± 10.43	**Physical well-being**	**Parent's View**
0.001	44.22 ± 11.77	40.59 ± 6.05	**Psychological well-being**	
0.000	49.51 ± 11.14	45.00 ± 4.29	**Autonomy and relationship with parents**	
0.024	46.52 ± 10.47	43.54 ± 10.71	**Social and peer relationship**	
0.000	51.09 ± 10.76	43.69 ± 8.21	**School environment**	

*P-value is reported for the Mann-Whitney test

**Table 3:** Comparison of the epileptic children’s and their parents' report on QOL items

** QOL items**	**Child **	**Parent **	P-value*
	**Mean ± SD**	** Mean ± SD**	
**Physical well-being**	50.94 ± 0.70	49.98 ± 10.43	0.041
**Psychological well-being**	41.92 ± 4.63	40.59 ± 6.05	0.460
**Autonomy and relationship with parents**	44.21 ± 4.01	45.00 ± 4.29	0.000
**Social and peers relationship**	41.18 ± 7.75	43.54 ± 10.71	0.000
**School environment**	46.40 ± 5.59	43.69 ± 8.21	0.071

**In Conclusion**

As stated previously, QOL is the self-perceived evaluation of QOL. Thus, proxy reports are not valid substitutes for personal perceptions of QOL. The present study is the first one on the QOL of epileptic children in Iran. According to the results obtained based on reports of both the children and parents, except the subscale related to physical health, the epileptic children showed significantly lower scores on the subscales of psychological well-being, relationship with parents, autonomy, social support, interaction with peers and finally school environment. It was also shown that patients experiencing more seizures per year had a lower QOL. However, patients receiving more drugs did not show a significantly lower QOL, possibly due to the better medical management of their disease. On the other hand, being a boy and receiving anticonvulsant drugs for a long period of time showed to have a worsening effect on the patients' QOL. It should be noted that QOL is a multi-factorial issue, and there is no standard and convergent agreement on what factors should be the center of attentions. Thus, since different studies have had variant approaches, comparison of the results cannot be highly straightforward. Moreover, this is the first study of its type conducted in Iran. Therefore, we lack a ground for proper comparison for of the results obtained in the study. Accordingly, there is a need to design other studies with larger population and also to further investigate other factors affecting QOL in order to have a more conclusive reslt.
